# Wiring miRNAs to pathways: a topological approach to integrate miRNA and mRNA expression profiles

**DOI:** 10.1093/nar/gku354

**Published:** 2014-05-06

**Authors:** Enrica Calura, Paolo Martini, Gabriele Sales, Luca Beltrame, Giovanna Chiorino, Maurizio D’Incalci, Sergio Marchini, Chiara Romualdi

**Affiliations:** 1 Department of Biology, University of Padova, via U. Bassi 58/B, 35121 Padova, Italy; 2 Department of Oncology, IRCCS Istituto di Ricerche Farmacologiche “Mario Negri”, via La Masa 19, 20145 Milan, Italy; 3 Cancer Genomics Laboratory, Fondazione Edo ed Elvo Tempia Valenta, via Malta 3, 13900 Biella, Italy

## Abstract

The production rate of gene expression data is nothing less than astounding. However, with the benefit of hindsight we can assert that, since we completely ignored the non-coding part of the transcriptome, we spent the last decade to study cell mechanisms having few data in our hands. In this scenario, microRNAs, which are key post-trascriptional regulators, deserve special attention. Given the state of knowledge about their biogenesis, mechanisms of action and the numerous experimentally validated target genes, miRNAs are also gradually appearing in the formal pathway representations such as KEGG and Reactome maps. However, the number of miRNAs annotated in pathway maps are very few and pathway analyses exploiting this new regulatory layer are still lacking. To fill these gaps, we present *‘micrographite*’ a new pipeline to perform topological pathway analysis integrating gene and miRNA expression profiles. Here, *micrographite* is used to study and dissect the epithelial ovarian cancer gene and miRNA transcriptome defining and validating a new regulatory circuit related to ovarian cancer histotype specificity.

## INTRODUCTION

In the last years, genome-wide expression studies of genes and microRNAs (miRNAs) have given a strong impulse in the comprehension of the cell regulatory mechanisms. Moreover, it has been increasingly clear that the integration of different *omic* data, although challenging, is a successful approach to have a wider perspective of the complexity of the biological systems.

Currently, miRNA and gene circuits are identified through the combination of binding prediction and expression correlation analyses (1–4). Although effective in many cases the simple correlation does not imply a causal relationship and a lot of false positive miRNA–mRNA interactions are still found. Moreover, miRNA and target genes are characterized by many-to-many relationships and they should be considered as part of a much more complex system of cellular interactions. On the contrary, correlation analyses are based on one-to-one relationships, one miRNA and one gene and they ignore the biological context of these cell signals.

Notwithstanding the defective analysis procedures, since their discovery, miRNAs have been extensively studied and hundreds of target genes have been found. However, despite all these efforts, signaling pathways that are the formal description of biological circuits, contain very few miRNAs. This penalizes pathway analyses, widely used and essential approaches to enhance transcriptome measurements interpretations.

To overcome this limitation we developed a new computational pipeline, called *micrographite*, able to integrate pathway information with predicted and validated miRNA–target interactions and to perform integrated topological analyses of miRNA and gene expression profiles to identify miRNA–gene circuits.

The advantage of our approach is two-fold: (i) it performs integrated pathway analyses to better describe the cellular scenario shaped by the underlying expression data, (ii) it selects and refines predicted miRNA–target interactions exploiting the biological context. With these features, *micrographite* is a helpful tool to guide the researcher in the interpretation of the expression measurements.

Here, we used our pipeline to study the regulatory circuits in the early stage of Epithelial Ovarian Cancer (EOC). EOC is the most common cause of death in gynecological diseases, with a 5-year survival rate virtually unchanged for the past 30 years (5,6). Despite the enormous effort which has been invested in understanding this malignancy, the causes of its pathogenesis are still unknown, as well as the mechanism of disease in the early phases of the carcinogenesis. In this context, a better characterization of the molecular events of EOC subtypes can be potentially helpful to clarify the mechanism of the disease and to improve diagnosis. In this paper, gene and miRNA expression profiles of 73 snap-frozen stage I EOC biopsies have been studied to understand the circuits that guide the mucinous histotype specificities. Additionally, real time-Polymerase Chain Reaction (PCR) expression measurements of a portion of the identified circuit were provided showing the reliability of the method.

## MATERIALS AND METHODS

### *micrographite* pipeline

#### Wiring miRNA to pathway

**Figure 1.**
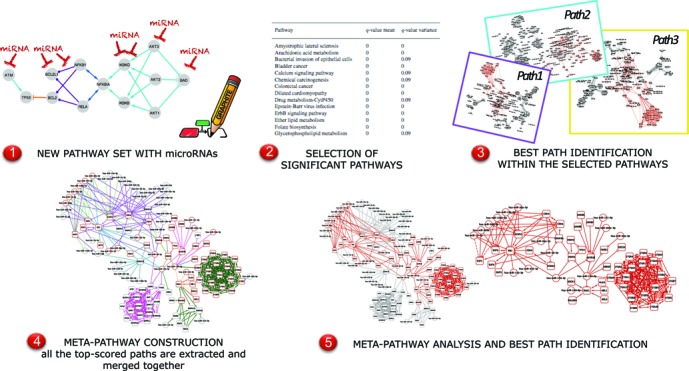
Graphical outline of the computational approach.

Pathway topologies derived from *graphite*, a Bioconductor package that we recently developed to store, manage and convert pathway annotations (from BioPax or KGML formats) into gene–gene networks. *graphite* is a pathway data interpreter that, following biologically driven rules, is able to solve the complexity of the pathway modules to generate interaction networks suitable for topological pathway analyses (7). The current version of *graphite* gathers pathway annotations from Kyoto Encyclopedia of Genes and Genomes (KEGG) (8), Reactome (9), Nature Pathway Interaction Database (NCI) (10) and Biocarta databases. *micrographite* expands a gene-based pathway graph adding a miRNA into the pathway if, and only if, at least one of its target gene is a node of the gene-based network (Figure 1, step 1). Two different batches of miRNA–target interactions are considered: the *in silico* predicted interactions and the validated ones. The validated miRNA–target interactions are derived from mirTarBase (11), miRecords (12) and a manual bibliographic research, selecting only the interactions validated by reporter assay. Due to the daily increase of the miRNA–target validated data, public databases are not exhaustive, thus the manual bibliographic research is a fundamental step to have updated biological information. We strongly encourage the users to manually update the list of validated targets at least for those miRNAs known to be related to the studied conditions. This step can be simplified using databases such as miRbase (13), miRWalk (14) or miR2Disease (15). As an example, we realized that some fundamental miRNA–target interactions validated on ovarian cancer were missing, such as some interactions involving miR-200 family. The predicted miRNA interactions are selected using prediction algorithms scores filtered by expression correlation analyses, therefore, the insertion of predicted miRNA–target interactions is dataset dependent. Although a miRNA has the role to down-regulate its target levels, the miRNA expression is not always anti-correlated with the target expression, in fact their relationship is dependent on the topology of the signaling pathway in which this interaction occurs (16). For this reason, in our setup, we consider miRNA–target with both correlated and anti-correlated interactions.

Specifically in the ovarian cancer dataset we used TargetScan predictions (with *P*_ct_ ≥ 0.8) and predicted miRNA–mRNA couples with a Pearson correlation coefficient |r| ≥ 0.4 and *q*-value ≤0.05. See Supplementary Material S1 for some statistics regarding the numbers of predicted and validated interactions included in the pipeline jointly with their overlap.

#### Topological pathway analysis

The topological pathway analyses used in *micrographite* is a modified version of *CliPPER*. *CliPPER* is a Bioconductor package that implements a topological pathway analysis based on Gaussian graphical model theory and then it is able to deal with data deriving from different sources with possibly different measurement scales. Given the topological structure of a pathway graph, the procedure implements a two-step strategy: (i) it selects pathways with covariance matrices or means significantly different between experimental conditions; and (ii) on these selected pathways, it identifies portions of the pathway (hereafter called 'paths') mostly associated with the phenotype. The identification of paths within a significantly altered pathway is one of the most innovative *CliPPER* features. Briefly, path identification is based on the graph decomposition into small-connected components, called cliques. Each clique is tested independently (according to the test on the means and/or concentration matrices) and then a significant level (*P*-value) for each clique is obtained. Following the Gaussian graphical model theory, maximal cliques can be used to reconstruct a junction tree. Junction tree is a hyper-tree having cliques as nodes and satisfying the running intersection property according to which, for any cliques *C*1 and *C*2 in the tree, every clique on the path connecting *C*1 and *C*2 contains *C*1 ∩ *C*2. Using the junction tree structure (that resembles the signal propagation of the pathway) we are able to identify and score all the paths in the graph. A path is a list of adjacent significant cliques (allowing a maximum of one gap, where a gap is a non-significant clique). The score of a path is a function of all the *P*-values of the cliques contributing to the path (significant *P*-value enhances the score, while a gap penalizes it); higher the score, better the path. For more technical and methodological details see (17).

Working pathway-by-pathway, the high degree of overlap of pathway annotations is not considered and this may lead to redundant results or truncated paths (a path that starts in a pathway and ends into another one) (18). To cope with these drawbacks *micrographite* implements a recursive procedure as follows: (i) *selection of pathways* – pathways are selected according to the significance levels obtained from the test on the mean or/and on concentration matrices of the whole pathway graphs (Figure 1, step 2); (ii) *best path identification*— for each of the previously selected pathways the path with the highest score is selected (Figure 1, step 3); (iii) *meta–pathway construction* — all the top-scored paths identified in the previous steps are combined generating a meta–pathway. The combination is the non-redundant union of all the top-scored paths, in an effort to reduce redundancy and to unify truncated paths (Figure 1, step 4) and (iv) meta–pathway analysis — the meta–pathway paths are analyzed and ranked according to their involvement in the phenotype (Figure 1, step 5).

### Implementation details

*micrographite* is available as R functions under the AGPL-3 license. Code and guideline about *micrographite* pipeline is available at http://romualdi.bio.unipd.it/micrographite. The latest version of Bioconductor, *graphite* and *CliPPER* packages are required. *micrographite* can be used with validated and/or predicted miRNA–target interactions. Depending on the amount of expression data analyzed, it could take several hours on an entire pathway database. Specifically, using an Intel(R) Xeon(R) 3.30GH processor, the presented analysis on ovarian cancer data with 10 000 permutations, using validated and predicted miRNA–mRNA interactions and the KEGG pathway database took 20 hours.

Permutation is used to estimate the significance levels of the test on the mean and on the concentration matrices either for the whole pathway or for the cliques. Under the null hypothesis the two groups have equal multivariate means (for the test on the means) and equal covariance matrices (for the test on the concentration matrices). Thus, using a classical permutational approach, we randomly permute samples across groups.

### Patient collection

A cohort of 151 snap-frozen tumor biopsies was collected from a frozen tissue bank, located at the Department of Oncology, IRCCS-Mario Negri Institute, Milano, Italy. Biopsies were collected from patients who underwent surgery for EOC at the Obstetrics and Gynaecology Department, San Gerardo Hospital, Monza, Italy between September 1992 and March 2010, as described by Marchini *et al.* (19). Tumor tissue samples, collected at the time of surgery, were sharp-dissected and snap frozen in liquid nitrogen within 15 min from resection, and then stored at −80°C. The patients underwent a complete staging procedure, according to the International Federation of Gynecological and Obstetrics criteria (FIGO). All biopsies selected for the study belonged to patients naive to chemotherapy and with diagnosis of stage I EOC. Tumor histological types were determined following World Health Organization (WHO) standards. A written informed consent was obtained from all the patients enrolled in the study, which has been performed following the Declaration of Helsinki set of principles. The local scientific ethical committee approved the collection and the use of tumor samples. Seventy-three patients of the entire cohort have been used to perform matched miRNA and mRNA expression profiles using microarrays. The array set of biopsies are composed of 15 mucinous, 23 serous, 19 endometrioid and 16 clear cell histotypes. The remaining 78 samples (27 mucinous, 22 serous, 22 endometrioid and seven clear cell histotypes) have been used as external and independent validation set.

### Gene and miRNA expression measurements

Frozen tissues specimens (30 mg) were homogenized in an TissueLyser LT (Qiagen, Milan, Italy) and total ribonucleicacid (RNA) purified using RNeasy Mini Kit isolation system (Qiagen), following manufacturers protocols. Total RNA concentration and proteins contamination were determined by Nanodrop spectrophotometer (Nanodrop Technologies, Ambion). Only samples with a RNA integrity number (RIN) larger than six and a Nanodrop A260:280 ratio between 1.8 and 2.1 were further processed and aliquots stored at −80°C until use. Array experiments were performed using standard procedures as previously published by (20). Briefly, 100 ng of total RNA was reverse transcribed into Cy3-labeled cRNA using LowInput QuickAmp labeling kit (Agilent Technologies, Palo Alto, CA, USA) and hybridized with a RNA labeling and hybridization kit according to the manufacturer's instructions (Agilent Technologies). miRNA extraction, labeling and hybridization using commercially available G4470B human miRNA Microarray kit (Agilent) were performed as previously described (20), raw data have been submitted to ArrayExpress (E-MTAB-1067). For gene expression measurements we used the commercially available G4851B human whole GE Microarray kit (SurePrint G3 Human Gene Expression 8 × 60K v2 Microarray Kit Agilent Technologies) which consists of 60K features printed in an 8-plex format (8 × 60 array). The arrays were washed and scanned with a laser confocal scanner (G2565B, Agilent Technologies) according to the manufacturer's instructions. mRNA microarrays underwent standard post-hybridization processing and the intensities of fluorescence were calculated by Feature Extraction software v11 (Agilent Technologies). Gene expression raw data have been submitted to ArrayExpress (E-MTAB-1814). Raw data (mRNA and miRNA) were pre-processed to filter out those probes with more than 40% of measurements below the signal-to-noise threshold. Filtered data were normalized using quantile normalization (21).

### qRT-PCR validation

Gene and miRNAs expression levels were validated by qRT-PCR on both training (array set) and validation set of patients. qRT-PCR have been performed using Sybr Green protocol (Qiagen, Milano, Italy) on an Applied Biosystems 7900HT instrument. Experiments were run in triplicate, using 384-well reaction plates in an automatic liquid handling station (epMotion 5075LH; Eppendorf, Milano, Italy). Raw data was generated with Sodium dodecylsulphate (SDS) Relative Quantification software (version 2.3; Ambion-ABI), data were normalized using the geometric mean of the four independent housekeeping controls (for miRNAs: RNU6B, SNORD61, SNORD72, SNORD68; for genes: ACTB, B2M, PPIA and HPRT1). Two-sided Student's *t*-test were used to verify among groups mean differences; *P*-value ≤0.05 was considered statistically significant.

## RESULTS AND DISCUSSION

### Pipeline overview

*micrographite* pipeline, briefly summarized in Figure 1, is based on the use of two bioinformatic tools that we have recently developed in the context of pathway analysis: *graphite* (7) and *CliPPER* (17).

The method starts combining pathway topology and miRNA–target gene interactions (see Figure 1 step 1). Then, using these new set of networks it applies a modified and recursive version of *CliPPER* analysis (Figure 1, steps 2–5 and 'Implementation Details' section) on matched gene and miRNA expressions profiles (17). The final outcomes are (i) a list of pathways composed of genes and miRNAs associated to the phenotype (Figure 1, step 2) and (ii) a list of scored circuits composed by nodes, both genes and miRNAs and interactions that are strictly involved in the biological problem studied (Figure 1, step 5). See 'Materials and Methods' section for details.

### Wiring miRNAs to pathway

Pathway analyses are manually annotated and derived by a long and careful process of curation produced by experts in the field. Thus, in *micrographite* we decide to be respectful of the pathway curation philosophy adding prevalently the miRNA–target interactions validated with reporter assays. Using this criterion we avoid the high-rate of false positives that characterize other experimental methods such as high-throughput techniques, as well as text mining guided by abstract co-occurrences. In addition, we decide to include the good and conserved TargetScan predictions filtered by expression correlation analysis. This approach leads to the selection of a small set of predicted interactions reducing false positives. Finally, we ended with 2118 validated interactions, 57 predicted interactions and six both predicted and validated (see Supplementary Material S1 for details).

However, since miRNA interactions are included in pathways only if the target gene is a pathway member, only a part of the collected interactions can be used. Respectively 1285, 1320, 1413 and 848 miRNA–target interactions are integrated in KEGG, Reactome, NCI and Biocarta pathways. In Table 1, the mean and median numbers of miRNAs, genes, miRNA–gene and gene–gene interactions per pathways are reported. As expected, the number of miRNAs integrated in pathway annotations is proportional to the dimension of the pathway; of consequence KEGG database, which is characterized by large pathways, contains also the pathways with the highest number of miRNAs. Table 2 reports the top 20 KEGG pathways ordered by the ratio between the number of miRNAs and the original size of the pathway (with only genes). The pathways with the highest number of inserted miRNAs are disease related pathways that in some cases quadrupled their original size. Accordingly, the top target genes ranked by the number of experimentally validated miRNAs are disease associated genes, such as CDKN1A (39 validated targets), VEGFA and BCL2. These results testify the dominant role of medical research in the field of miRNA target discovery.

**Table 1.** Summary statistics of pathway composition after the integration of validated miRNAs

Database	No. of pathways	No. of miRNAs per pathway^a^	No. of miRNA–gene interactions per pathway^a^	No. of genes per pathway^a^	No. of gene–gene interactions per pathway^a^
KEGG	220	34.71 (18)	52.03 (20)	89.89 (59)	329.58 (162)
Reactome	1210	17.25 (6)	23.47 (6)	59.50 (26)	844.19 (95)
Biocarta	247	18.47 (13)	30.21 (25)	21.43 (14)	39.00 (28)
Nci	782	15.06 (5)	18.28 (5)	30.35 (14)	187.70 (24)

^a^Mean (median) numbers.

**Table 2.** Summary statistics of the top 20 KEGG integrated pathways ranked by the ratio between no. of miRNAs and no. of genes

Pathway name	No. of genes	No. of miRNAs + genes	$\frac{{\textrm {No.}}\, {\textrm {of}}\, \textrm {miRNAs}}{{\textrm {No.}}\, {\textrm {of}}\,\textrm {genes}}$
Bladder cancer	29	127	4.3
Viral carcinogenesis	6	26	4.3
Dorso-ventral axis formation	12	42	3.5
Rheumatoid arthritis	19	65	3.4
Malaria	11	35	3.1
Glioma	65	189	2.9
Thyroid cancer	28	80	2.8
Melanoma	69	196	2.8
Colorectal cancer	49	138	2.8
Chronic myeloid leukemia	73	204	2.7
Endometrial cancer	45	124	2.7
Pancreatic cancer	69	189	2.7
p53 signaling pathway	68	181	2.6
Non-small cell lung cancer	52	138	2.6
Prostate cancer	87	230	2.6
Renal cell carcinoma	60	145	2.4
Acute myeloid leukemia	57	133	2.3
ErbB signaling pathway	88	197	2.2
Small cell lung cancer	83	181	2.1
VEGF signaling pathway	67	146	2.1

### Epithelial ovarian cancer

The ovarian cancer can be divided in at least 15 types of tumors, each of them characterized by specific histopatological features, molecular alterations, risk factors, different chemotherapy responses and resistances. However, the main classification criterion is in histologic subtypes. Low-grade serous, mucinous, endometrioid and clear cell histotypes represent the great majority of stage I ovarian cancers. Given this scenario, the identification of subtype-specific biomarkers and the understanding of mechanisms that characterize the tumors might allow the development of more efficient diagnosis and treatment strategies. EOC is characterized by alterations of the epidermal growth factor receptor (EGFR), PI3K/AKT/mTOR signaling and by mutations or epigenetic losses of BRCA1/2, PTEN and TP53 (22,23). EOC shows loss of E-cadherin expression compared to epithelial cells resulting in a decreased cell–cell adhesion and nuclear localization of beta-catenin, which further promotes the invasive phenotype. The TGF-beta/SMAD signaling is one of the best candidate pathway involved in the aberrant regulation of the epithelial to mesenchymal transition in EOC (24,25). Using *micrographite*, we focused on the cell signaling characterization of mucinous histotypes. Mucinous ovarian carcinomas (MucEOC) is one of the four main subtypes of EOCs mostly diagnosed at early stages. MucEOCs develop almost always within the ovary and histologically it is indistinguishable from the mucinous non-ovarian carcinoma or the metastatic carcinoma with mucinous differentiation (cervix, colon/rectum, appendix cancers) (26). The numerous commonalities between ovarian and non-ovarian mucinous carcinoma, such as colorectal cancer have driven hypotheses about a shared aetiology and a possible common treatment of the two tumors. MucEOC is characterized by high frequencies of KRAS and BRAF mutations and ERBB2 amplifications. The pathway of mitogen activated protein kinase (MAPK), the WNT (18% of tumors) and TP53 pathways are altered (26). Moreover, mucEOC is characterized by high levels of miR-192 and miR-194, as well as mucinous non-ovarian cancer samples (20).

### *micrographite* results: the mucinous pathway

Using EOC dataset, KEGG pathways were enriched by 1285 validated miRNA–target interactions and 63 TargetsScan predictions (Supplementary Material S1). TargetScan predictions were selected using a correlation-based cut-off (|r|≥0.4). We tried different correlation thresholds (Supplementary Material S4) in order to test the robustness of the approach. Our results show that the inclusion of a large number of miRNA–target interactions (e.g. using a loose threshold such as |r|≥0.2) gives worse results than including lower but well refined interactions. This is in agreement with the idea that the inclusion of noise in the graph reduces the efficiency of the method hiding real biological signals.

Comparing mucinous versus non-mucinous samples we obtained a list of 22 significant pathways (*micrographite* step 2) reported in Table 3. All the pathways showing *P*-values ≤0.1 for both mean and variance tests were further considered for the follow analysis steps. The results at the pathway level are coherent with the biological knowledge characterizing mucinous subtype: the similarity of MucEOC with colorectal cancer, the involvement of the epidermal and transforming growth factors signals, such as the ErbB and the TGF-beta signaling and, moreover, the p53 signaling pathway. Also, the involvement of 'One carbon pool by folate' metabolic pathway is interesting in the light of the registered association between the high-risk of colorectal carcinoma and the folate depletion, which affects the mechanisms of DNA methylation, integrity and repair (27). Moreover, it is worth to note that melanoma and bladder cancer pathways share genes such as PI3K, TP53, MDM2, ERBB2, E-caderin, that seem to be associated also to MucEOC.

**Table 3.** Pathway level analysis. Pathway with adjusted *P*-value ≤0.1 for both mean variance tests are reported

Pathway name	*q*-value mean	*q*-value variance
Amyotrophic lateral sclerosis (ALS)	0	0
Arachidonic acid metabolism	0	0
Bacterial invasion of epithelial cells	0	0.09
Bladder cancer	0	0
Calcium signaling pathway	0	0.09
Chemical carcinogenesis	0	0.09
Colorectal cancer	0	0
Dilated cardiomyopathy	0	0
Drug metabolism - cytochrome P450	0	0.09
Epstein-Barr virus infection	0	0
ErbB signaling pathway	0	0
Ether lipid metabolism	0	0
Folate biosynthesis	0	0
Glycerophospholipid metabolism	0	0.09
Hypertrophic cardiomyopathy (HCM)	0	0
Melanoma	0	0.09
Metabolism of xenobiotics by cytochrome P450	0	0.09
One carbon pool by folate	0	0
p53 signaling pathway	0	0
TGF-beta signaling pathway	0	0
Type II diabetes mellitus	0	0
Viral myocarditis	0	0

**Figure 2.**
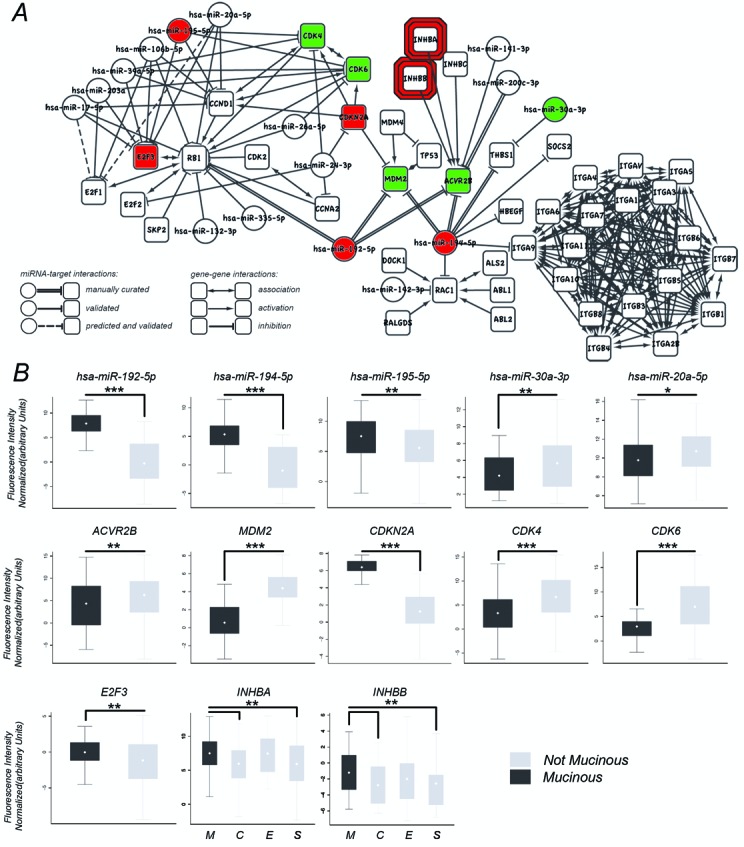
Mucinous ovarian cancer circuit and qRT-PCR validations. **(A)** The path with the maximum score: the mucinous path. As described in the legend, miRNA–target validated interactions can be achieved from both manual curation (literature mining) and public databases. Predicted interactions are obtained from Targetscan predictions filtered by expression correlations. **(B)** qRT-PCR boxplot for specific genes and miRNA of the best path. Mucinous (M), clear cell (C), serous (S), endometrioid (E): ***≤0.005, **≤0.05, *≤0.1.

All the significant pathways listed in Table 3 were analyzed looking for the best scored path inside each pathway (portion of the whole pathway see Supplementary Material S2). All the main paths of each pathway were then combined into a new non-redundant meta–pathway (Supplementary Material S3). As mentioned before, this path combination reduces redundant information due to pathway overlaps and connects disjoint paths due to the artificial subdivision of cellular processes into discrete entities (paths that start in a pathway and end in another one). The union of all the paths has produced two separated networks (Supplementary Material S4 Figure 1), in which the color of edges represents the pathways of origin. The meta–pathway was then analyzed in order to identify and rank its paths (See Supplementary Material S4 Figure 2 and Supplementary Material S5). This procedure allows the finding of chains of genes and miRNAs that are differentially involved in the mucinous compared to other histotypes. Figure 2A shows the meta–pathways that contains the maximum scored path hereafter called the mucEOC pathway.

The mucEOC pathway, composed of 61 elements (45 genes and 16 miRNAs), contains many EOC and MucEOC related genes, such as miR-192 and miR-194 that have a mucEOC expression specificity. Interestingly, in this representation of the cell signaling it is clear that miR-192 and miR-194 act as bridges across multiple cellular signals of ovarian cancer interest. Specifically, miR-192 controls the signaling of RB1/E2Fs and, together with miR-194, the MDM2/TP53 signaling. Both the signaling paths, RB1/E2Fs and MDM2/TP53, are controllers of cell cycle that seems to be deeply affected as suggested by the presence in the mucEOC pathway of cyclins (CCND1, CCNA2), cyclin-dependent kinase (CDK2, CDK4, CDK6) and cyclin-dependent kinase inhibitors (CDKN2A) (28–30). The signaling involving CDKN2A, MDM2, TP53, miR-192/194, has already been studied in detail for the characterization of the mucEOC (20). Moreover, in the mucEOC pathway, both miR-192 and miR-194, together with miR-200c and miR-141, regulate ACVR2B, a growth and differentiation factor which belongs to the transforming growth factor-beta (TGF-beta) superfamily. Both miR-200c and miR-141 are miRNA associated to EOC survival (19,31) together with the TGF-beta signaling that, acting on cell growth and differentiation, is considered crucial for the understanding of EOC molecular mechanism (32). In this context, the results of mucEOC pathway suggest an interplay of miR-192, miR-194, miR-200c and miR-141 in the down-regulation of ACVR2B, this scenario is already observed in other type of cancers associated with an aberrant regulation of the TGF-beta pathway (33). Then, miR-194 regulates both THBS1, the gene for the Thrombospondin, and ITGA9, a gene coding for an Integrin. These two proteins, being part of a system to regulate the cell-to-cell and cell-to-matrix interactions, are key elements to regulate apoptosis, cell proliferation and angiogenesis and, moreover, THBS1 is a gene already associated to EOC (34,35). Another interesting relation is the regulation of RAC1 operated by miR-194. In fact, RAC1, together with RALGDS, represents the signal downstream KRAS, an important gene that is known to be mutated and involved in mucEOC. KRAS mutations are deeply investigated for colorectal cancer, which is the pathway of origin of these interactions (36,37). Finally, two miRNA–target gene interactions of the mucEOC pathway are part of the initial batch of miRNA–target relations inserted in the pathways because *in silico* predicted couples with correlated expressions. These two *in silico* predictions are also already validated interactions (38), suggesting that *micrographite* could be used also to efficiently select the predicted miRNA–target couples for the assay validations.

### *micrographite* results using only gene expression: gaining power from miRNAs

The inclusion of miRNAs within a pathway analysis framework is by itself a great improvement since at the moment there are no methods able to topologically combine mRNA and miRNA within regulatory signals. However, to quantify this enhancement we performed *micrographite* pipeline using only gene expression data. The meta–pathway identified by the pipeline using only mRNA data is smaller and it is completely contained in the meta–pathway obtained using mRNA and miRNA datasets. The final best path obtained by mRNA data, is contained in the sixth path (ranked by max score) of the analysis using both mRNA and miRNAs (see Supplementary Material S6). These findings further suggest that the inclusion of miRNAs improves and expands the gene analysis results. This is not a surprising conclusion at least for two reasons: from a methodological point of view, the more complete the pathways, the more accurate the analysis; from an applicative point of view, miRNAs are known to play a key role in tissue specificity and in regulation of cancer circuits (39–41). Thus, at least for our case study, miRNA inclusion in the pathway analyses is a necessary and a major improvement.

### Expression validations

In addition to numerous literature confirmations on the accuracy of our results, we performed some additional measurements by qRT-PCR. For the validation, we focused on miR-192 and miR-194 and their action on the circuit upstream p53 composed by CDKN2A and MDM2, plus some elements closed to this circuit (CDK6, CDK4, E2F3, ACVR2B, INHBB,INHBA, miR-195-5p, miR-20a-5p, miR-30a) for a total of eight genes and five miRNAs. Specifically, the expression of miR-192/194 cluster is directly controlled by wild type TP53 that, enhancing their transcriptions, is able to arrest cell cycle (42). Among the targets of miR-192/194 is MDM2, a negative regulator of TP53 (43). These relationships define a positive feed back loop involving TP53 that, through miR-192/194, inhibits its own inhibitor. Another interesting element linked to this circuit is CDKN2A (44), which prevents the degradation and inactivation of p53 operated by MDM2 (45). All qRT-PCRs on these genes confirm the differential involvement of this circuit in MucEOC samples in respect to the other EOC histotypes (Figure 2C and Supplementary Material S7). Specifically, miR-192, miR-194 and CDKN2A results up-regulated in MucEOC and their target MDM2 down-regulated. Furthermore, ACVR2B, CDK4, CDK6, E2F3, miR-195-5p, miR-20a-5p and miR-30a were differentially expressed in mucinous versus all the other histotypes, while INHBA, INHBB show expression levels significantly higher in mucinous with respect only to serous and clear cell samples. These results confirm the involvement of this regulatory circuit in the characterization of mucinous subtype.

## CONCLUSION

The *micrographite* pipeline allows integrated analyses of gene and miRNA expression profiles within a pathway-based context. At our knowledge this is the first attempt in this direction. We applied successfully *micrographite* to dissect the complexity of regulatory networks in epithelial ovarian cancer. The circuit obtained conforms to the existing knowledge and adds new insights for the comprehension of the mucEOC subtype.

For researchers working with miRNAs, the advantages of our approach are several: (i) the possibility to integrate and (ii) analyze miRNA and mRNA expression profiles using pathway information and (iii) to biologically contextualize miRNA–mRNA validated and predicted interactions. The *micrographite* results allow the biologist to have a clear and more complete overview of cell behavior, this is fundamental in the understanding of cellular processes and to formulate new hypotheses during the interpretation of expression data.
